# A novel bioluminescent herpes simplex virus 1 for in vivo monitoring of herpes simplex encephalitis

**DOI:** 10.1038/s41598-021-98047-z

**Published:** 2021-09-21

**Authors:** Olus Uyar, Pier-Luc Plante, Jocelyne Piret, Marie-Christine Venable, Julie Carbonneau, Jacques Corbeil, Guy Boivin

**Affiliations:** 1 Research Center in Infectious Diseases, CHU de Québec-Laval University Research Center and Department of Pediatrics and Microbiology, Faculty of Medicine, Laval University, Quebec City, QC Canada; 2 Research Center in Infectious Diseases, CHU de Québec-Laval University Research Center and Department of Molecular Medicine and Big Data Research Centre, Faculty of Medicine, Laval University, Quebec City, QC Canada

**Keywords:** Herpes virus, Viral pathogenesis, Viral infection

## Abstract

Herpes simplex virus 1 (HSV-1) is responsible for herpes simplex virus encephalitis (HSE), associated with a 70% mortality rate in the absence of treatment. Despite intravenous treatment with acyclovir, mortality remains significant, highlighting the need for new anti-herpetic agents. Herein, we describe a novel neurovirulent recombinant HSV-1 (rHSV-1), expressing the fluorescent tdTomato and Gaussia luciferase (Gluc) enzyme, generated by the Clustered regularly interspaced short palindromic repeats (CRISPR)—CRISPR-associated protein 9 (Cas9) (CRISPR-Cas9) system. The Gluc activity measured in the cell culture supernatant was correlated (P = 0.0001) with infectious particles, allowing in vitro monitoring of viral replication kinetics. A significant correlation was also found between brain viral titers and Gluc activity in plasma (R^2^ = 0.8510, P < 0.0001) collected from BALB/c mice infected intranasally with rHSV-1. Furthermore, evaluation of valacyclovir (VACV) treatment of HSE could also be performed by analyzing Gluc activity in mouse plasma samples. Finally, it was also possible to study rHSV-1 dissemination and additionally to estimate brain viral titers by in vivo imaging system (IVIS). The new rHSV-1 with reporter proteins is not only as a powerful tool for in vitro and in vivo antiviral screening, but can also be used for studying different aspects of HSE pathogenesis.

## Introduction

Herpes simplex virus 1 (HSV-1), a member of the *Alphaherpesvirinae* subfamily, is a neurotropic virus composed of approximately 150 kb of linear dsDNA^1^. This neurotropic virus infects more than 60% of the world population and establishes lifelong latent infection in sensory neurons, mainly in trigeminal ganglia^2^. Although most infections appear to be asymptomatic or mild, HSV-1 infection can also result in life-threatening conditions such as herpes simplex virus encephalitis (HSE) and neonatal infections^3^. HSE is an uncommon neurological infection, while it remains the most frequent lethal sporadic acute viral encephalitis worldwide. Despite acyclovir therapy, the mortality rate of patients with HSE is approximately 30%^4^. Moreover, most surviving patients still suffer from significant neurologic impairments because of neuronal and glial damage caused by viral replication and subsequent inflammation. It is also known that the permeability of the blood–brain barrier (BBB) is altered during HSE, leading to progressive neuroinflammation that contributes to neuropathological sequelae^5^.

HSE studies in mice usually require the sacrifice of animals at specific time-points to harvest the brain that will be used for histological or molecular analyses^6^. However, these methods based on sequential sacrifices prevent longitudinal observations of infected animals. Recombinant HSV-1, engineered by different genome-editing strategies, such as bacterial artificial chromosome (BAC) recombination, allowed to identify viral gene functions of HSV-1 and characterize mutations affecting the pathophysiology of HSE. Unfortunately, genomic engineering of clinical strains is highly complex, and many of these recombinant viruses exhibited attenuated virulence in animal models^7^. Recently, Clustered regularly interspaced short palindromic repeats (CRISPR)—CRISPR-associated protein 9 (Cas9) (CRISPR-Cas9) system was adapted for DNA editing in mammalian cells, which makes genomic manipulation of large viral DNA sequences easier and more efficient^8^. This new method first induces a specific double-strand DNA break (DSB) by an RNA-guided nuclease, then enables genome editing by repairing DSB via either error-prone non-homologous end joining (NHEJ) or homology-directed repair (HDR). Several recombinant herpes viruses, including HSV-1, were produced using the CRISPR-Cas9 system and can be used to conduct virus-host interaction studies^9,10^.

Recombinant pathogens expressing different types of luciferase have enabled the monitoring of infections by in vivo bioluminescence imaging (BLI). This non-invasive imaging method allows real-time visualization of central nervous system (CNS) infections on a single animal throughout the course of infection^11^. Moreover, the emerging use of the CRISPR-Cas9 system made the generation of recombinant viruses expressing reporter proteins easier. The latter approach enabled observing the dissemination of neurotropic viruses into the CNS and identifying anatomic regions of the brain where high levels of viral replication occur^12^. Despite these recent advances, the application of BLI in HSE studies remains limited.

This study describes the generation and characterization of a novel recombinant herpes simplex virus 1 (rHSV-1) designed as a BLI tool to monitor HSE in living mice. Neurovirulent rHSV-1 was created by inserting two reporter genes coding for tdTomato fluorescent protein and Gaussia Luciferase (Gluc) enzyme, into the UL26-UL27 intergenic region of clinical HSV-1 strain H25, via Cas9-guide RNA (gRNA)-induced HDR strategy. After isolation of rHSV-1, the recombination event was validated by PCR and whole genome sequencing (WGS). Further, we studied in vitro and in vivo correlations between brain viral titers and relative light units (RLU) emission from different biological samples. As a proof-of-concept, we first evaluated the effect of valacyclovir treatment in HSE by analyzing Gluc activity in mouse plasma. Secondly, we used the newly generated rHSV-1 to study viral dissemination into the CNS and examine the BBB integrity by BLI. Here, we propose that rHSV-1 is a useful tool for in vitro and in vivo antiviral screening and for studying HSE pathogenesis.

## Results

### Generation and isolation of recombinant HSV-1

**Figure 1:**
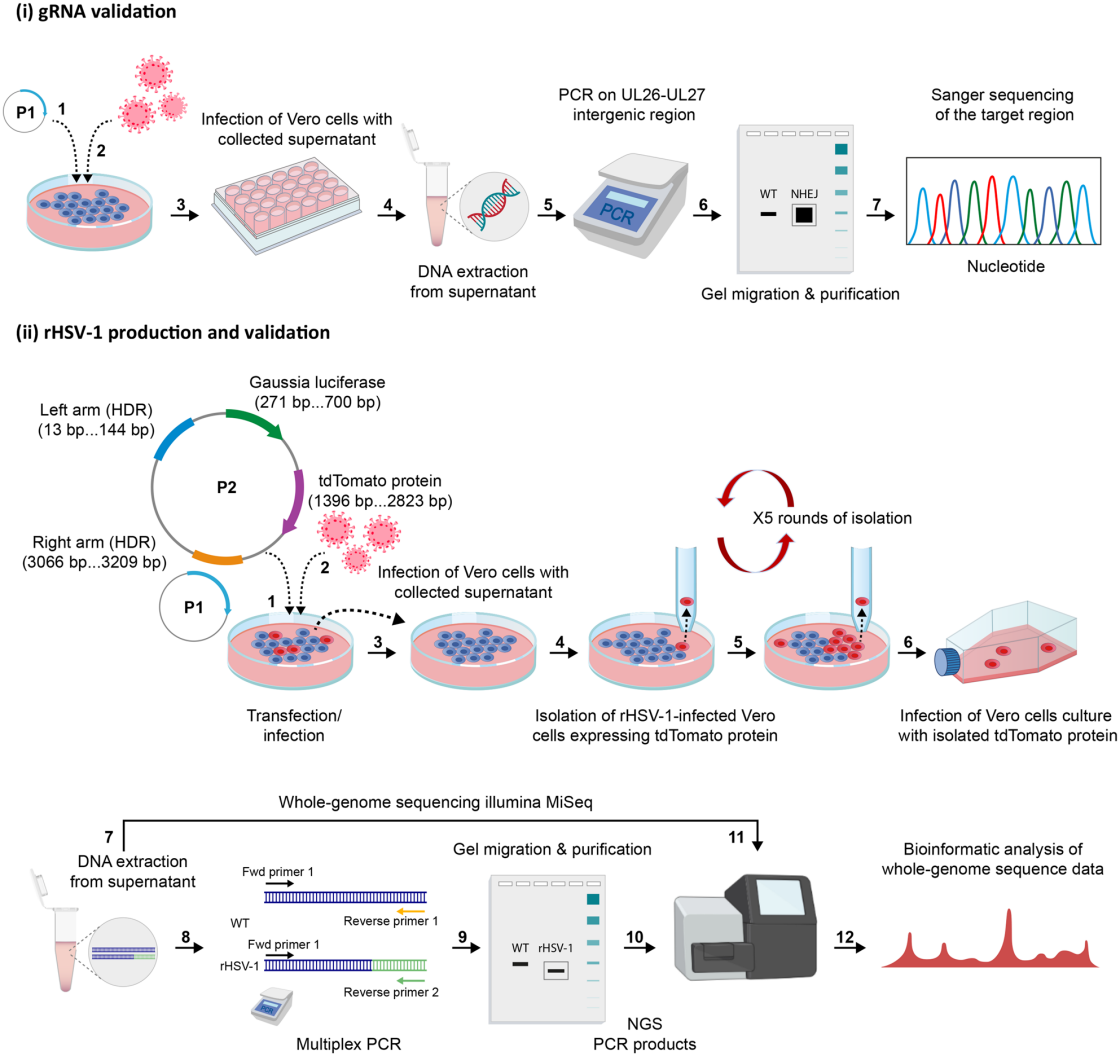
CRISPR-Cas9-mediated generation of recombinant HSV-1 (rHSV-1). Schematic presentation of two workflows: (**i**) gRNA validation and (**ii**) rHSV-1 production and the validation. (**i**) gRNA validation: Following the transfection/infection step (P1: CRISPR-Cas9 vector/ HSV-1 strain H25), collected supernatants were diluted and used to infect naïve Vero cells. Supernatant was collected and viral DNA was amplified. PCR products from the amplification of UL26-UL27 intergenic region were first gel-purified, then Sanger sequenced for NHEJ-mediated indel mutation analysis. (**ii**) rHSV-1 production and validation: Following the transfection/infection step (a mixture of plasmids (P1: CRISPR-Cas9 vector and P2: The HDR (homology-directed repair) donor plasmid)/HSV-1 H25 strain), collected supernatants were used to infect naïve Vero cells. tdTomato^+^ rHSV-1 infected cells were isolated and used to infect naïve Vero cells. Five rounds of isolation/infection process were performed to eliminate all WT HSV-1 virions from rHSV-1 viral production. Supernatant was collected and DNA extraction was performed. PCR products from multiplex PCR were gel-purified. Two viral DNAs (WT HSV-1 H25 strain, rHSV-1) and gel-purified Multiplex PCR products were deep-sequenced by using Illumina MiSeq. Bioinformatic analysis was performed for whole-genome construction of both viruses. Images were created using Adobe Illustrator (version 25.3.1/https://www.adobe.com/).

The transfection/infection approach was performed to generate rHSV-1 expressing Gluc and tdTomato reporter proteins, using the CRISPR-Cas9 system^13^. Briefly, this method consists of transfecting host cells with a mixture of CRISPR-Cas9/gRNA and the donor plasmid prior to their infection with the virus to modify (Fig. 1, two workflows: (i) gRNA selection and (ii) rHSV-1 production and validation). We designed three gRNAs (Table 1) to induce the viral DNA cleavage in the UL26-UL27 non-coding intergenic region, which was already well-characterized^13,14^. In silico analysis on CHOPCHOP suggested a more significant efficiency (61.23%) for gRNA-2 to create the DSB.

**Table 1:** DNA oligonucleotides used to construct gRNA plasmids.

gRNAs in silico efficiency (CHOPCHOP)	Target sequences/target regions*	Oligo-1/Oligo-2**
gRNA-1/57.04%	AGGAAAGAGGAAACAGGCCG*CGG*/UL26-UL27	5′-*caccg*AGGAAAGAGGAAAACAGGCCG-3′ 5′-*aaac*CGGCCTGTTTCCTCTTTCCTC-3′
gRNA-2/61.23%	GGAATCGGCACTGACCAAGG*GGG*/UL26-UL27	5′-*caccg*GGAATCGGCACTGACCAAGG-3′ 5′-*aaac*CCTTGGTCAGTGCCGATTCC-3′
gRNA-3/57.7%	GGGGAATCGGCACTGACCAA*GGG*/UL26-UL27	5′-*caccg*GGGGAATCGGCACTGACCAA-3′ 5′-*aaac*TTGGTCAGTGCCGATTCCCC-3′
gRNA-4/0%	GTCCATATTTGTGCTGTGCCG*CGG*/UL29-UL31	5′-*caccg*GTCCATATTTGTGCTGTGCCG-3′ 5′-*aaac*CGGCACAGCACAAATATGGAC-3′

*gRNA sequences (20 nucleotides) followed by PAM-3′ (italics capital letters).

**5′-caccg (Oligo-1) and 5′-aaac (Oligo-2) (italics small letters) overhangs were added to gRNA oligos to clone these oligos into CRISPR vector.

**Figure 2:**
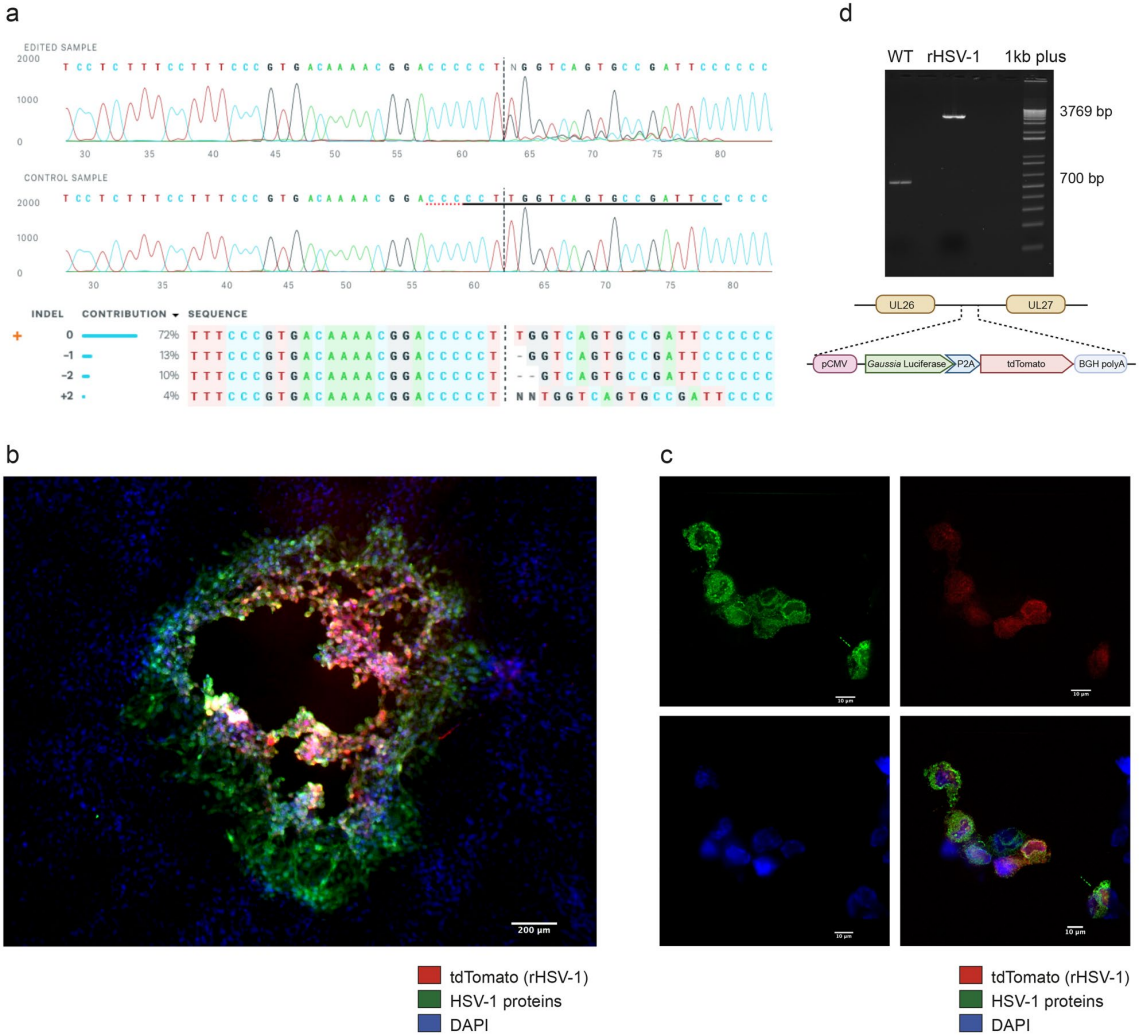
rHSV-1 production, isolation, and validation process (**a**) Validation of gRNA-2 targeting UL26-UL27 intergenic region. Sanger sequencing chromatograms of UL26-UL27 PCR amplicons from edited samples containing WT and recombinant HSV-1 show heterozygous peaks between 64 and 80th nucleotides that correspond to NHEJ-mediated indel introduction after gRNA-2 guided Cas9 cut (top). The horizontal black and red dotted-underlined regions represent gRNA and PAM sequences, respectively. Example of Inference of CRISPR Edits’ (ICE) analysis on a gRNA-2-edited viral clone showing indel mutations in UL26-UL27 intergenic region of HSV-1 (bottom). The contributions show the inferred sequences present in the edited population and their relative proportions. Black vertical dashed lines represent cut sites (top and bottom) and the WT sequence is indicated with a “+” on the left. (**b**) The isolation of rHSV-1 was confirmed by immunostaining analysis of infected Vero cells for rHSV-1. Immunofluorescence analysis of infectious spots (scale bar: 10 μm, magnification 5X) showed that all infectious spots were tdTomato^+^ (red) and HSV-1^+^ (green). (**c**) Confirmation of tdTomato expression by rHSV-1^+^ Vero cells. tdTomato expression (red) was confirmed by confocal imaging of infected Vero cells labeled with HSV-1 proteins (green) and counterstained with DAPI (blue) (scale bar: 10 μm, magnification 100 ×). Following the selection process of rHSV-1, Vero cells on cover slips were infected with rHSV-1, then washed, fixed, permeabilized, blocked and immunostained. (**d**) Confirmation (top) and schematic presentation of the incorporation of exogenous fragment (bottom) into UL26-UL27 intergenic region. Agarose gel electrophoresis of PCR products obtained from the UL26-UL27 intergenic region amplification of WT HSV-1 (700 bp) and rHSV-1 (3796 bp).

Heterozygous peaks between 64 and 80th nucleotides of PCR amplicons corresponding to NHEJ-mediated repair into the gRNA-2 target site were observed on Sanger sequencing chromatogram (Fig. 2a, top). Based on our initial observations, we assumed that gRNA-2 modified-viral pool contained at least 15 to 20% of mutant HSV-1^15,16^. In addition, the indel analysis on the UL26-UL27 amplicons of mutant HSV-1 showed that mutation frequencies were higher in gRNA-2 edited HSV-1 fragments (highest indel frequency for gRNA-2: 27%) compared to other gRNA target sites (Fig. 2a, bottom).

Next, the exogenous fragment carrying reporter genes, with flanking 128 pb left and 150 pb right homology arms, was introduced into the virus genome by HDR of CRISPR-Cas9-induced DSB. Two reporter genes under the control of cytomegalovirus (CMV) major immediate-early (MIE) promoter were connected with 2A sequence coding for a self-cleaving peptide. This allows the co-expression of two reporter proteins from a single mRNA^17^. The open reading frame (ORF) was adjusted by the addition of two nucleotides (-TA), and the bovine growth hormone (bgh) polyadenylation signal was placed downstream from the tdTomato gene and its stop codon. Moreover, we introduced a protospacer adjacent motif (PAM) altering-mutation that changed –GGG to –GGA to avoid re-cutting the recombinant virus genome, as reported elsewhere (Supplementary Fig. 1)^18^. The progeny virus was identified, and a single HSV-1 tdTomato^+^ plaque was isolated under a fluorescence microscope (Fig. 2b). Further, tdTomato expression was evaluated in rHSV-1-infected cells by a confocal microscope. Infected Vero cells were immunostained with an antibody anti-HSV-1 proteins (Fig. 2c). Finally, we confirmed by PCR that the whole insert was present in the UL26-UL27 intergenic region of isolated rHSV-1 (Fig. 2d).

### Whole-genome characterization of the recombinant HSV-1

**Figure 3:**
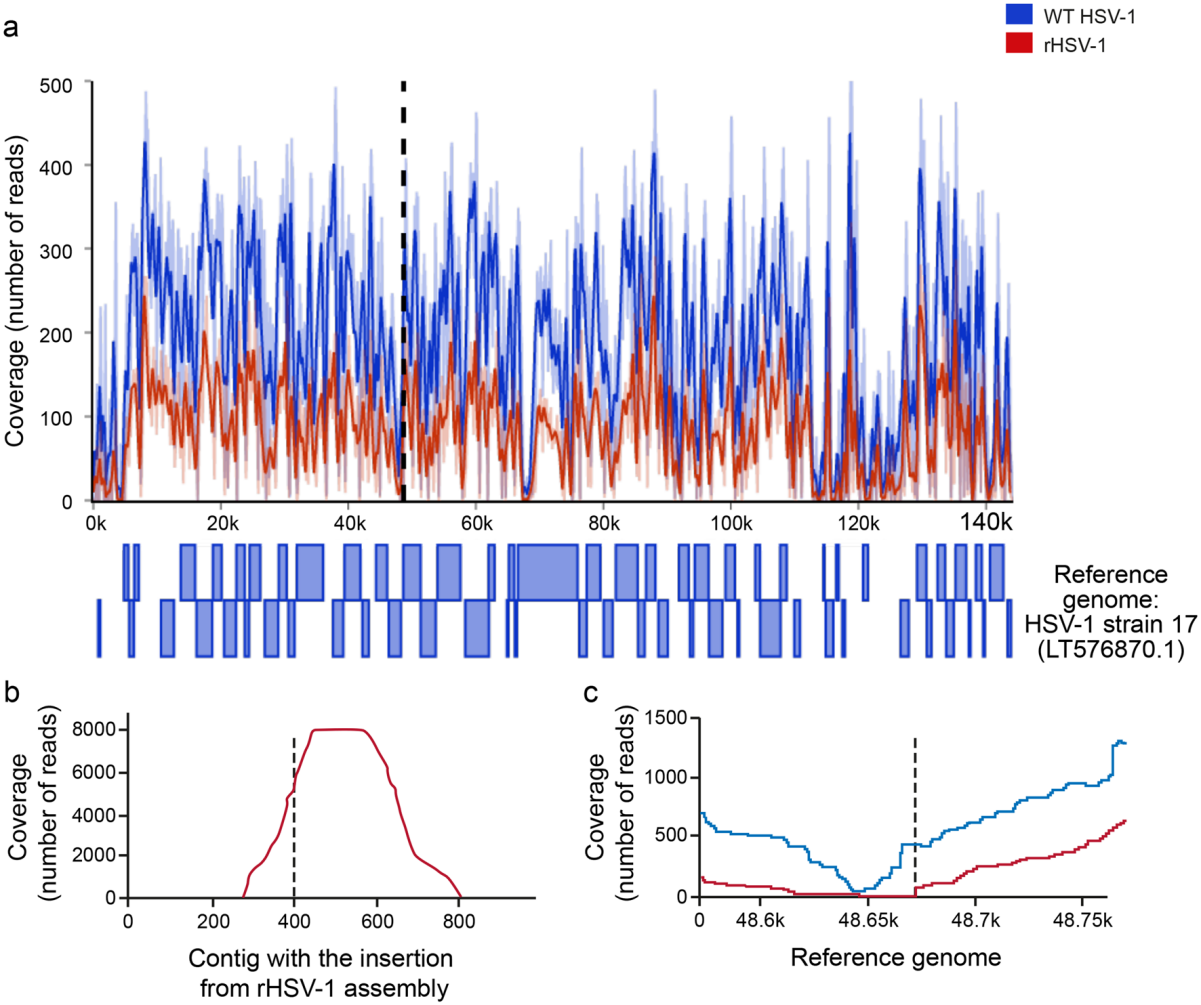
Sequencing depth (Coverage (number of reads) from high-throughput sequencing of the whole genome and the insertion junction. (**a**) Average sequencing depth over 500 bp. after alignment of HSV-1 strain H25 (blue) and recombinant HSV-1 (rHSV-1) (red) on reference genome of HSV-1 strain 17. The dash line represents the expected insertion junction. (**b**) Sequencing depth of the multiplexed PCR library over the contig from rHSV-1 that contains the insertion. The PCR clearly shows that the expected junction is amplified. (**c**) Zoomed view from (**a**) to better compare the sequencing depth at the expected insertion position from HSV-1 strain 17. There are no reads from rHSV-1 that overlap the insertion point but coverage increases right after, suggesting a successful insertion.

The mutation acquired during the generation/isolation process of rHSV-1 may alter the viral phenotype. Thus, we analyzed the whole genomes of wild-type (WT) HSV-1 and rHSV-1 by WGS. As previously shown, WGS and assembly of HSV-1 is highly challenging due to high GC content and multiple repeat sequences^19^. To this end, Sanger sequences were used in combination with Illumina short reads. Overall, sequencing depth varied from 1 to over 300 in some regions when aligning reads to HSV-1 strain 17 reference genome (Fig. 3a). Most coding regions were fully covered, allowing to perform variant calling. Overall, 438 variations were found between strain H25 and strain 17 coding sequences. The variations were dispersed in most regions, except for the sequences of UL45 to ICP0, which mainly remained unchanged.

Analysis of the multiplexed PCR assay showed a variable coverage between 1447 and 7997, 100 bp before and after the expected position of the insertion, with a depth of 5756 × on the insertion junction (Fig. 3b). Comparatively, no reads from the PCR assay aligned to HSV-1 strain 17 genome. Similar results were observed, but with a lower sequencing depth, when using WGS data and aligning the reads on HSV-1 strain 17 genome (Fig. 3c).

Variant calling of rHSV-1 on WT HSV-1 de novo assembled contigs showed 12 single nucleotide polymorphisms (SNPs), three of them resulting in a substitution, five being silent mutations on coding regions and four mutations on small contigs that could not be aligned to a coding region. The three missense substitutions appeared in three different proteins (UL29, UL38 and UL41) during the generation and isolation process of rHSV-1. Val361Ile (position on reference genome: 56,727) substitution was found in UL29 encoding ICP8 that plays a crucial role in viral DNA replication. ICP8 promotes helicase activity of helicase-primase complex (UL5-UL8-UL52) of HSV-1. The residues between 332 to 564 of ICP8 were claimed to be involved in DNA binding^20^. UL38 gene, encoding triplex capsid protein VP19C, had a Leu156Val substitution (position on reference genome: 80,699)^21^. Random insertions occurring in the central region of VP19C can affect viral growth^22^. A Thr461Ile substitution (position on reference genome: 88,254) was detected in UL41 encoding for virion host shutoff (vhs) protein. This viral RNase suppresses all cellular protein synthesis during HSV-1 infection^23^. It is known that vhs may help HSV-1 evade immune responses by inhibiting mechanisms such as cyclic GMP-AMP synthase-stimulator of interferon genes (cGAS/STING) interferon-β activation pathway^24^. This newly identified substitution is located in region III (amino acids 365 to 489) of UL41 polypeptide, where mutations could potentially enhance viral replication^25^.

At last, gRNA off-target activity was analyzed to understand if these mutations could be induced by CRISPR-Cas9 system. First results on CHOPCHOP with a closely related HSV-1 genome (HSV-1 strain 17, GenBank: JN555585.1) showed no possible off-target for selected gRNAs. Further in silico analysis by searching homology between gRNAs and WT WGS reads also confirmed that no off-target modification was possible using these gRNAs.

### Introduction of reporter genes into UL26-UL27 intergenic region did not alter in vitro viral replicative capacity

**Figure 4:**
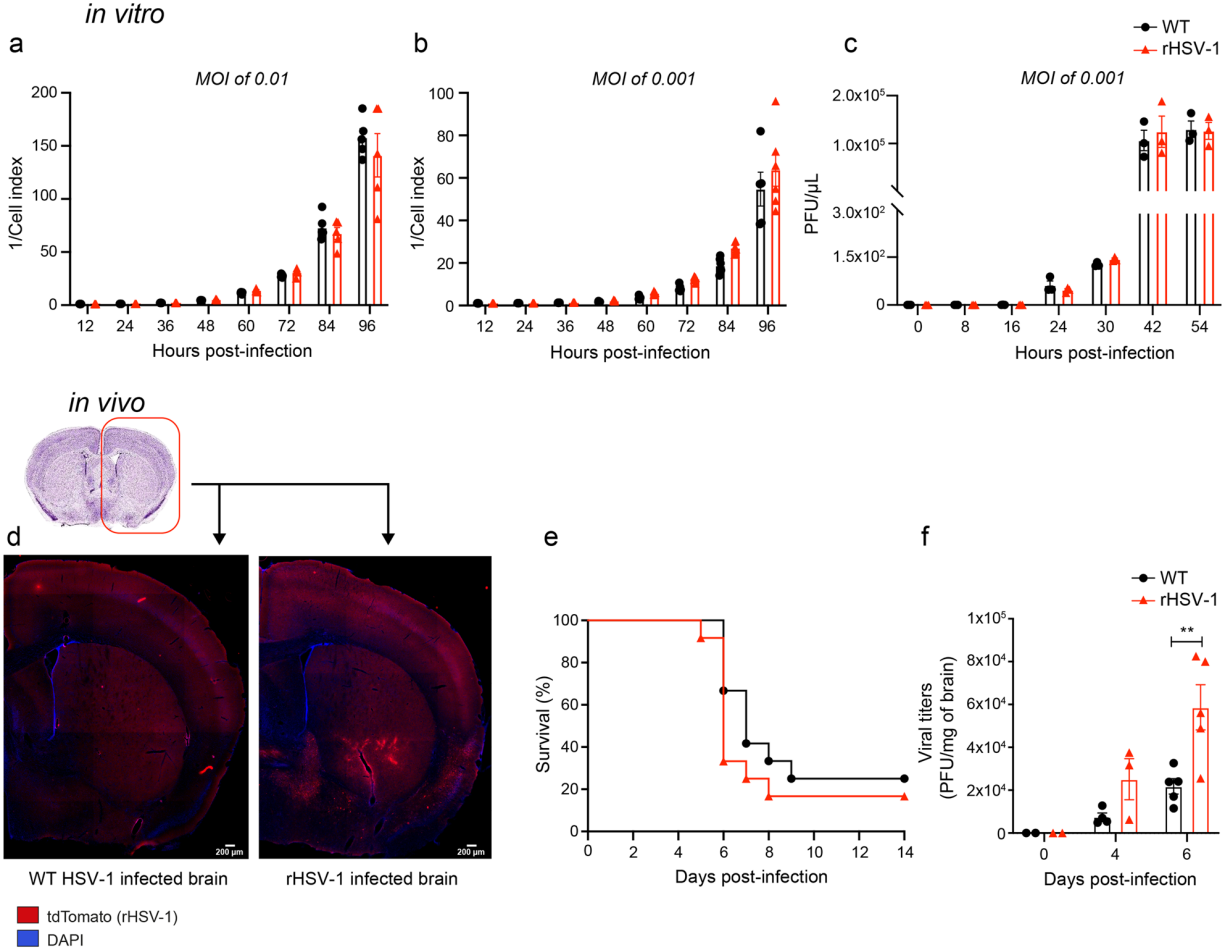
In vitro and in vivo replicative capacities of rHSV-1 (**a**, **b**) Replicative capacity of wild-type (WT) HSV-1 and recombinant HSV-1 (rHSV-1) were determined by real-time cell analysis (RTCA) for 96 h. Two different multiplicity of infection (MOI) [0.01 (**a**) and 0.001 (**b**)] were used to infect Vero cells. Data are represented as 1/cell index, a dimensionless parameter that reflects changes of cellular morphology or growth rate (WT: black bars and circles, rHSV-1: red bars and triangles). (**c**) Replicative capacity of wild type (WT: HSV-1 strain H25) and recombinant HSV-1 (rHSV-1) virus at a MOI of 0.001, as determined by plaque assay. Vero cells were infected with both viral strains for 90 min at 37 °C in a 5% CO_2_ atmosphere. The viral suspensions were removed, and cells were incubated with MEM plus 2% FBS. The cell culture supernatants were collected for 54 h. Results represent the means ± SEM for triplicate. (**d**) Representative fluorescent microscopy images of a WT HSV-1 (left) and rHSV-1-infected (red) (right) brain sections counterstained with DAPI (blue) on day 6 post-infection. tdTomato signal is localized in the bed nuclei of stria terminalis and medial preoptic area. (**e**) Survival curves of mice infected with WT HSV-1 (black line with circles) and rHSV-1 (red line and triangles). Survival rates were analyzed using a log-rank (Mantel-Cox) test. (**f**) Viral titers in brain homogenates were measured by a standard plaque assay on Vero cells on days 0, 4 and 6 post-infection. The results are reported as PFU per milligram of brain homogenates and represent the means ± SEM for 3 to 4 mice per group at each time point. Statistical analyses were performed using two-way ANOVA with Sidak's multiple comparisons test.

The replicative capacity of the WT and rHSV-1 strains was evaluated in Vero cells by real-time cell analysis (RTCA) and confirmed by plaque assay. Two different multiplicity of infection (MOIs) of 0.01 and 0.001 were used to infect cells, and the cell index (CI) was recorded for 4 days. We found that the viral replicative capacity of rHSV-1 remained unchanged compared to WT HSV-1 for both MOIs (Fig. 4a,b). We confirmed our data by comparing the replication capabilities of WT and rHSV-1 by plaque assay. Once again, we observed similar trends in replication kinetics (Fig. 4c) of both viruses, suggesting that neither the large foreign genes in the intergenic region of UL26-UL27 nor previously described SNPs in the rHSV-1 genome seem to have a significant impact on in vitro replication properties.

### Enhanced neurovirulence of rHSV-1 does not result in higher mortality rates

We then examined whether rHSV-1 remained as neurotropic as the WT HSV-1 strain H25 after genomic modifications. To evaluate the neurovirulence of rHSV-1, we infected BALB/c mice intranasally with 1 500 plaque forming unit (PFU) of WT or rHSV-1. On day 6 post-infection (p.i.), brain sections stained for HSV-1 proteins were analyzed by immunofluorescence microscopy. The detection of tdTomato^+^ CNS cells on rHSV-1-infected brain sections proved that rHSV-1 was able to infect the CNS (Fig. 4d). This observation was also validated by colocalized signals of tdTomato (rHSV-1) and GFP (HSV-1 proteins) on rHSV-1 infected CNS cells (Supplementary Fig. 2a). Although we observed lower tdTomato^+^ spots than those of GFP^+^, there was no difference in distribution patterns of infectious spots in CNS of rHSV-1- or WT HSV-1-infected mice (Supplementary Fig. 2b).

We further studied mortality rates and in vivo replicative kinetics of rHSV-1 in BALB/c mice infected intranasally with 1 500 PFU of WT or rHSV-1. We observed similar mortality rates for both strains (Fig. 4e, 75% for WT HSV-1 *vs* 83.4% for rHSV-1, P-value: 0.29, non-significant). Viral titers in the CNS of rHSV-1-infected mice were 2.7 times higher than those infected with WT HSV-1 (Fig. 4f, 21,808 PFU/mg in WT brains, 58 643 PFU/mg in rHSV-1; P-value: 0.003) on day 6 p.i. These results suggest that, although rHSV-1 was associated with increased in vivo replicative capacity compared to WT, the two strains had similar mortality rates.

### In vitro and in vivo viral titers correlate with secreted Gluc activity

**Figure 5:**
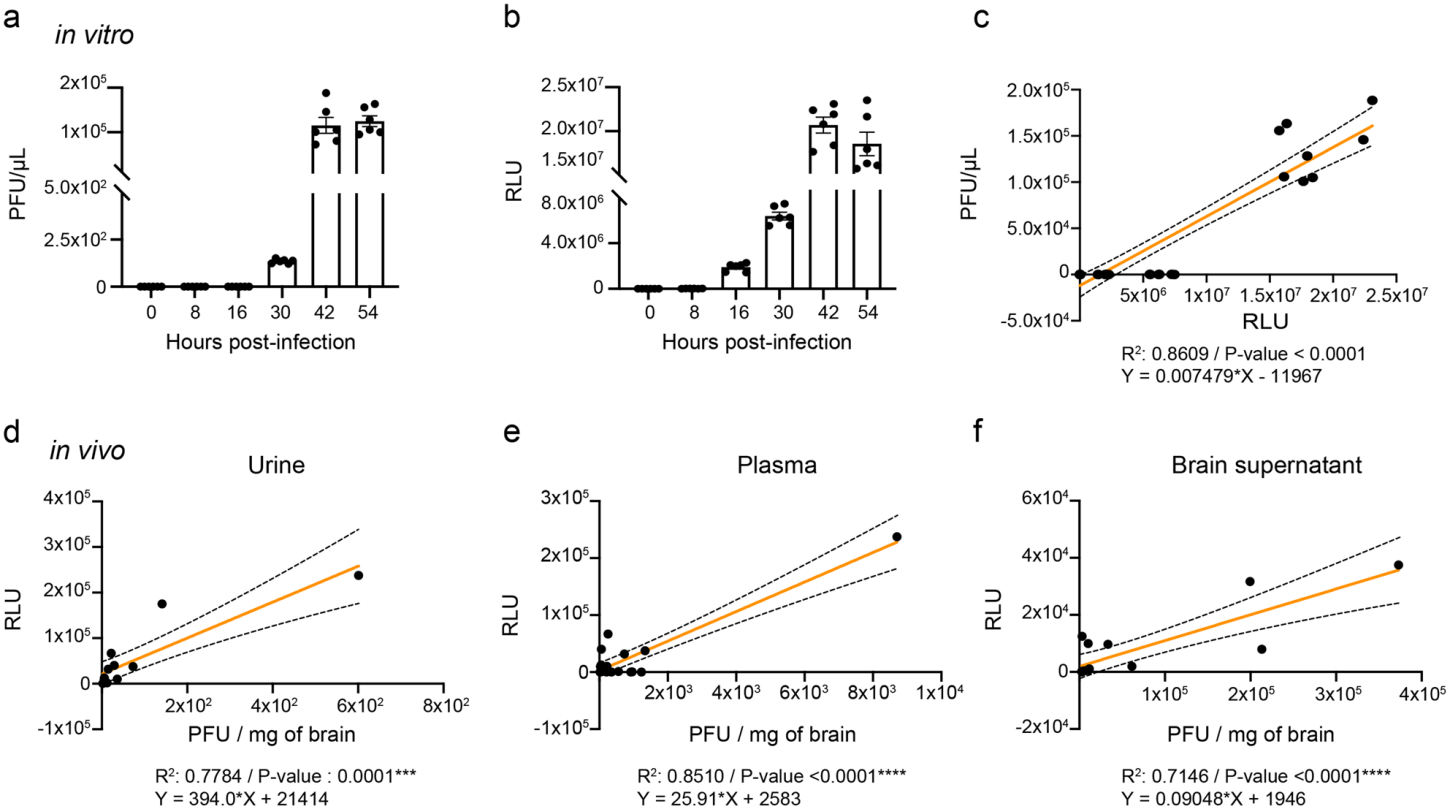
In vitro and in vivo relative light units (RLUs) and rHSV-1 titers correlations. (**a**) Replicative capacity of recombinant HSV-1 (rHSV-1) at a MOI of 0.001 as determined by plaque assay. Vero cells were infected with rHSV-1 for 90 min at 37 °C in a 5% CO_2_ atmosphere. The viral suspensions were removed, and cells were incubated with MEM complemented with 2% FBS. The cell culture supernatants were collected for 54 h. (**b**) Gluc secretion was detected in vitro in supernatants. Gluc in supernatants were analyzed for relative light units (RLU). Results are presented as mean ± SEM. (**c**) Previously measured virus titers and corresponding RLU values were analyzed with a simple linear regression (linear regression line presented in orange). The regression equation, confidence intervals (black dashed lines), R^2^ and P-value are shown in the graph. Six-week-old BALB/c male mice were infected intranasally with 1 500 PFU of HSV-1 strain H25 in 20 μL of minimal essential medium. Brain, blood and urine samples were collected on days 0, 4 and 6 post-infection. rHSV-1 titers in brains and Gluc activities in urine (**d**), plasma (**e**) and brain homogenate supernatants (**f**) were analyzed with a simple linear regression. The regression equation, confidence intervals (dashed lines), R^2^ and P-value are shown in the graph.

Standard viral titration techniques are time-consuming and labor-intensive. To overcome these limitations, we determined whether the activity of secreted Gluc was correlated with the growth kinetics of rHSV-1. We measured cell culture supernatant viral titers by plaque assay (Fig. 5a), then performed a Gluc activity assay on those supernatants (Fig. 5b). As expected, Gluc activity was observed only in cells infected with rHSV-1. Significant RLU values were detected as early as 8 h post-infection (h p.i.) and reached a maximum at 42 h p.i. with more than 80% of Vero cells infected by rHSV-1. Significant correlations were observed between Gluc activity and PFUs (Fig. 5c, R^2^ = 0.86, P-value < 0.0001) in cell culture supernatants. Overall, these data suggest that rHSV-1 in vitro replication can be monitored by Gluc assay.

Furthermore, we measured ex vivo Gluc activities in brain, blood and urine samples collected from rHSV-1-infected mice prior to and on days 4 and 6 p.i. A correlation study between viral titers in the CNS and RLUs obtained from the same samples was conducted. Positive correlation between brain viral titers and Gluc activity in mouse samples was observed for urine, plasma, and brain supernatant samples (Fig. 5d, urine: R^2^ = 0.7784, P-value: 0.0001; Fig. 5e, plasma: R^2^ = 0.8510, P-value < 0.0001; Fig. 5f, brain supernatant: R^2^ = 0.7146, P-value < 0.0001). However, we did not observe any correlation between Gluc activity in whole blood and viral titers in the CNS, suggesting an incompatibility of the Gluc test with whole blood. These results indicate that an accurate estimate of viral titers in the CNS could be performed by Gluc assay in readily accessible samples such as plasma and urine.

### Rapid assessment of antiviral compounds against HSE by measuring luciferase activity in plasma samples of mice

We first evaluated the effective acyclovir concentration that reduced the cytopathic effects by 50% (EC50s) for both WT and rHSV-1. The EC50 of acyclovir against WT and rHSV-1 strains in Vero cells were, respectively, 1.567 ± 0.306 μg/mL and 0.947 ± 0.129 μg/mL by plaque reduction assay (PRA). Based on a 1.65-fold decrease in EC50s of rHSV-1 (compared to EC50s of WT HSV-1; P-value: 0.1, non-significant), we concluded that there was no significant change in acyclovir susceptibility between these two viruses.

**Figure 6:**
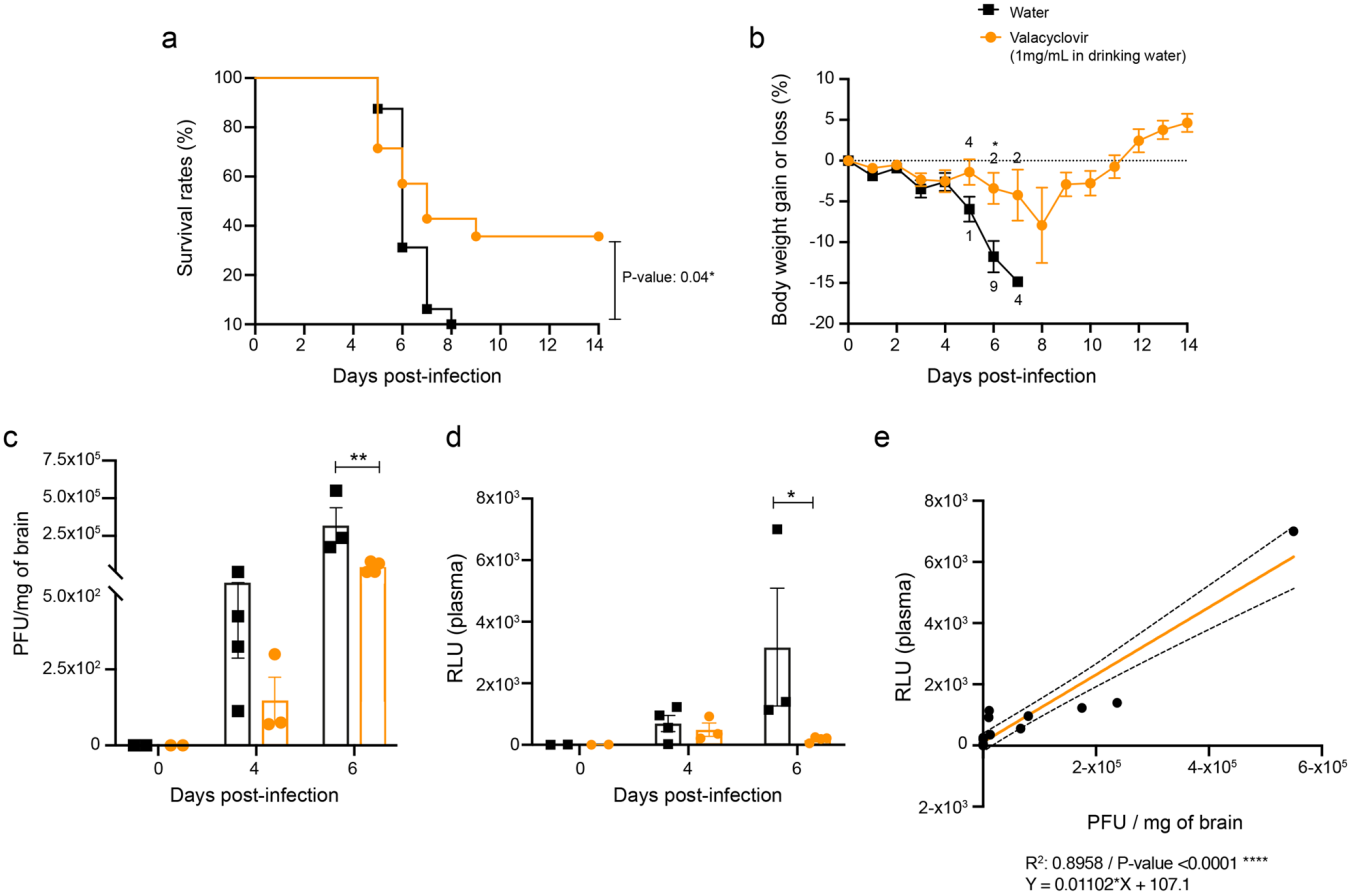
Luciferase activity-based rapid assessment of valacyclovir treatment against HSE. The effect of valacyclovir (VACV) administration was studied in 6-week -old male BALB/c mice infected intranasally with 2500 PFU of HSV-1 strain H25 in 20 μL of minimal essential medium. Infected mice were treated with VACV (1 mg/mL in drinking water) from day 3 post-infection and on. The differences in survival rates (**a**) and percentage of body weight changes (**b**) were evaluated for 14 days post-infection, using Log-Rank (Mantel– Cox) and Holm-Sidak multiple comparison t-tests, respectively. The digits along the curves (**b**) correspond to the numbers of dead mice on the indicated day. Viral titers in the CNS (PFU/mg of brain) (**c**) and luminescence (RLU) (**d**) in plasma samples of infected mice were measured on days 0, 4 and 6 post-infection. Results represent the means ± SEM. Statistical differences between groups (Control group: black squares or bars; VACV: orange circles or bars) were obtained using two-way ANOVA with Sidak's multiple comparisons test. rHSV-1 titers in brains and Gluc activities in plasma (**e**) were analyzed with a simple linear regression. The regression equation, confidence intervals (black dashed lines), R^2^ and P-value are shown in the graph.

The poor outcome of HSE despite acyclovir therapy underlines the need for new antiviral and anti-inflammatory compounds. We suggest that our in vivo antiviral drug testing method based on plasma Gluc activity, correlating with viral titers in the CNS, may accelerate the evaluation of new drugs. As a proof of concept, we assessed the effect of valacyclovir (VACV; a prodrug of acyclovir) on viral titers by measuring plasma Gluc activity on days 0, 4 and 6 p.i. We infected BALB/c mice intranasally with 2 500 PFU of rHSV-1 and treated them with VACV (1 mg/mL in drinking water ad libitum) from day 3 p.i. and on, as described elsewhere^26^. The effect of valacyclovir on weight and survival rate of infected mice was evaluated for 14 days. VACV-treated mice exhibited a significant increase in survival rate compared to control mice that did not receive VACV (Fig. 6a, 35% for VACV, 0% for control; P-value: 0.04). In addition, mice that received VACV demonstrated significantly lower body weight loss than control mice on days 5 and 6 p.i. (Fig. 6b, P-value: 0.038 and 0.003, respectively). Finally, the impact of VACV on viral titers in CNS was investigated. We observed a 20-fold increase in viral titers in the CNS of control mice on day 4 p.i. (compared to VACV-treated mice; P-value > 0.99, non-significant). Viral titers were significantly lower in brain homogenates of VACV-treated mice on day 6 p.i. (Fig. 6c, 320 833 PFU/mg of brain in control vs 42 291.8 PFU/mg of brain in VACV-treated mice; P-value 0.0029).

We then evaluated the activity of Gluc in plasma samples at different time points to assess whether RLU kinetics follow the same trend as viral titers in the CNS. As expected, plasma RLU values of VACV-treated animals were significantly lower on day 6 p.i., compared to those from the control group (Fig. 6d, P-value: 0.04, 178.75 RLU for VACV group vs 3 178 RLU for the control group). Once again, we validated the positive correlation between RLUs obtained from plasma samples and viral titers in the CNS. (Fig. 6e, R^2^ = 0.8958, P-value < 0.0001). Altogether, our data suggest that plasma Gluc activity correlates with viral titers in the CNS during an antiviral intervention.

### Real-time monitoring of rHSV-1-induced HSE

**Figure 7:**
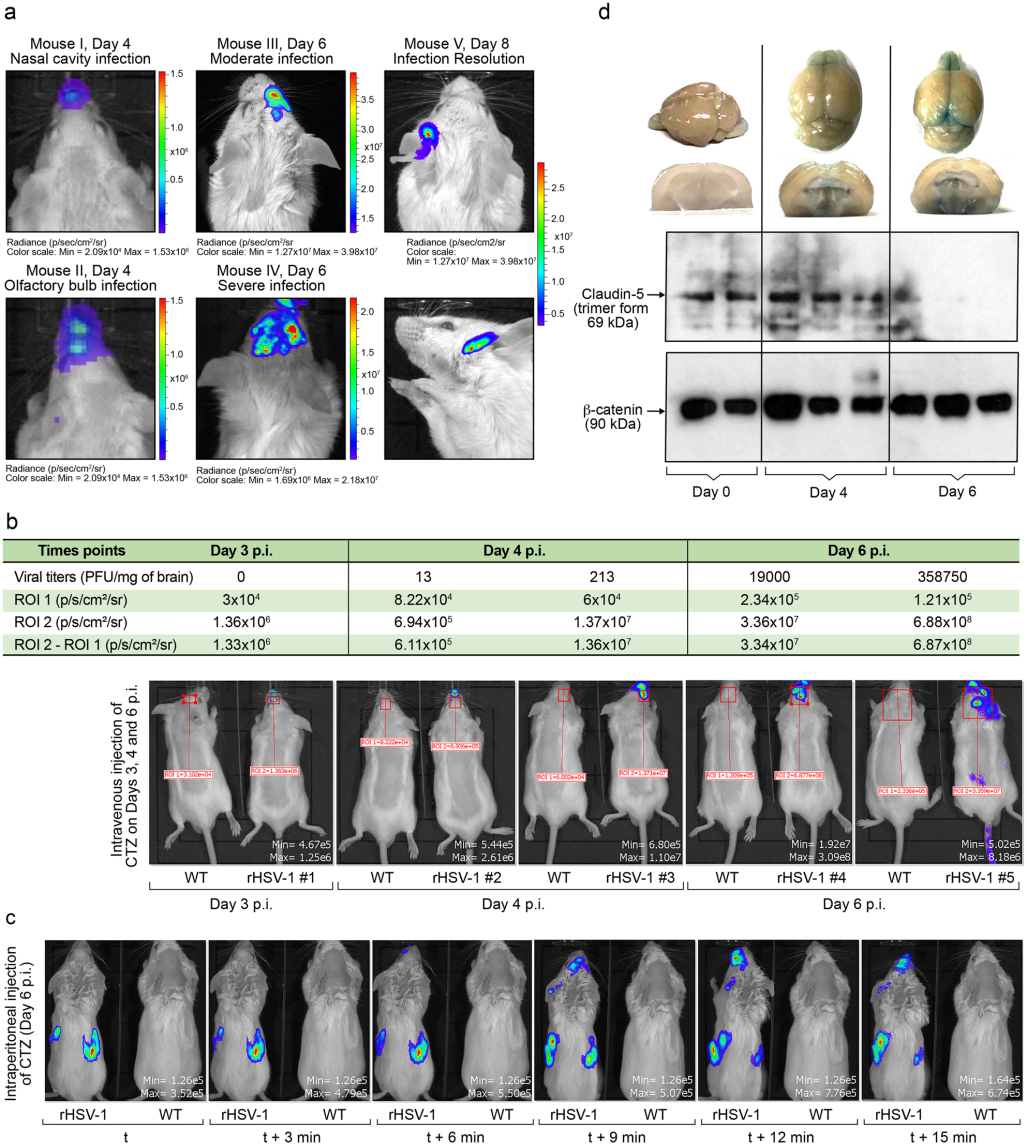
Study of viral dissemination in the CNS and viral titer estimation in herpes simplex virus encephalitis (HSE) by bioluminescent imaging (BLI). (**a**) Representative BLI images of early (Mouse I and Mouse II, Day 4), moderate (Mouse III, Day 6) and severe (Mouse IV, Day 6) HSE are shown at different time points. Two images acquired from different angles (both images captured from the same “Mouse V, Day 8”) allowed a better anatomical localization of infected areas. (**b**) A bioluminescence imaging series of representative mice infected with 1 500 PFU of WT HSV-1 (mice on the left side) or rHSV-1 (mice on the right side) on days 3, 4 and 6 p.i. The bioluminescent signal is expressed in average radiance (p/s/cm^2^/sr). Animals infected with rHSV-1 were sacrificed and brains were removed to measure viral titers by plaque assay. The above table shows ROI-1 (negative control area, mouse infected with WT HSV-1 strain H25), ROI-2 (bioluminescent signal in the CNS, mouse infected with rHSV-1) radiance values (p/s/cm2/sr), ROI-2–ROI-1 (normalized bioluminescent signal) and viral titers in brains for each rHSV-1 infected-mouse (**c**) Time-course imaging of rHSV-1-infected mice to evaluate blood–brain barrier (BBB). Following intraperitoneal CTZ injection, mice inoculated with 1 500 PFU of rHSV-1 were imaged every 3 min on day 6 post-infection. (**d**) BBB permeability is increased in rHSV-1-infected brains. Whole brain photos show that olfactory bulbs (on days 4 and 6 p.i.) and superior colliculus (on day 6) were intensely stained with extravasated Evans Blue dye (EBD). Coronal sections of whole brains showed EBD accumulation in thalamus and periventricular nucleus in hypothalamus of brains on days 4 and 6 p.i. (above). Western blots for claudin-5 were performed with whole brain extracts from rHSV-1-infected mouse brains. Trimer forms of Claudin-5 (69 kDa) expression were highly reduced on day 6 p.i.

To detect the presence of rHSV-1 in vivo, we inoculated BALB/c mice intranasally with 1 500 PFU of rHSV-1 or WT HSV-1. Following intravenous injection of coelenterazine (CTZ), we could visualize bioluminescence signals produced by Gluc activity in the CNS of rHSV-1-infected mice on days 4, 6 and 8 p.i. (Fig. 7a). As expected, there was no signal detected in mice infected with WT HSV-1.

The time course of rHSV-1 dissemination from the nasal cavity into the CNS parenchyma was investigated by analyzing various rHSV-1-infected BALB/c mouse images from different time points. We noticed that the first signal in the CNS appeared on day 4 p.i. in olfactory bulbs, corresponding to the early stage of the disease (Fig. 7b—rHSV-1#2). On days 4 and 6 p.i., an intense signal emerged from the eye of some infected mice (Fig. 7b, rHSV-1#3, rHSV-1#4, rHSV-1#5). Simultaneously, the diencephalon and several spots in the mesencephalon were detected (Fig. 7b, rHSV-1#4 and rHSV-1#5). BLI on day 8 p.i. also revealed an infectious site in an area that we assume to be the piriform cortex, a part of the central olfactory tract (Fig. 7a, Mouse V, Day 8: Infection Resolution). In addition, immunofluorescence (IF) studies on HSV-1-infected brain sections showed that optic tract, olfactory tubercule, bed nuclei of stria terminalis and paraventricular hypothalamic nucleus were the main infected areas on day 6 p.i. (Supplementary Fig. 2b,c). Overall, we suggest that HSV-1 uses the olfactory pathway and visual circuitry to disseminate into the thalamus and hypothalamus^27^.

Next, we evaluated whether IVIS-detected bioluminescence intensity [total flux (p/s) in manually defined Regions of Interest (ROIs)] emitted from the CNS correlates with brain viral titers (Fig. 7b). The correlation study conducted on five animals at different time points (Fig. 7b, table) suggested a strong correlation (R^2^: 0.9996, P-value < 0.0001, not shown) between these two parameters. Our result demonstrates the potential of BLI to monitor viral titers in the CNS simply by measuring bioluminescent signals on IVIS-acquired images.

### BLI of rHSV-1-infected mice reveals blood–brain barrier damage during HSE

The BBB is a highly specialized structure formed by microvascular endothelial cells joined by tight junctions. CNS infections by a neurotropic virus may disrupt the BBB. It has been shown that neither Gluc nor its substrate, CTZ, can cross the BBB^28,29^. Our objective was to evaluate whether infection with HSV-1 alters the integrity of the BBB by BLI.

We injected i.p. CTZ to rHSV-1 infected mice on day 6 p.i. Following the CTZ administration, multiple image acquisitions (with an interval of 3 min) on the same mouse revealed the bioavailability of Gluc in different organs. First, signals appeared from i.p. injection site to the kidney and spleen (Fig. 7c, blue spots on image t). However, no signal was emitted from the CNS until the 6^th^ min (Fig. 7c, image t + 6 min). Signals from the right side of the mouse body started to quench with CTZ diffusing into the systemic circulation^30^. We noticed that some CNS regions, including olfactory bulbs, diencephalon, and eyes, began to emit signals during this shift. As expected, no signal emerged from control mice infected with WT strain (Fig. 7c, mouse on the right side). Altogether, we speculate that CNS infection by HSV-1 leads to a dysfunctional BBB, allowing CTZ to diffuse into the brain parenchyma and Gluc to accumulate in different organs.

To confirm BBB disruption, we used intravenous Evans Blue dye (EBD) injection in rHSV-1-infected mice on day 6 p.i. to visualize dye extravasation into the brain parenchyma. Following the i.v. EBD injection, we harvested the brain to observe if EBD could cross BBB (Fig. 7d, top). Our observations on day 4 p.i. indicate that BBB disruption occurred in olfactory bulbs. On day 6 p.i., the olfactory bulbs were intensively stained with EBD. We also noticed that the BBB disruption progressed towards the mesencephalon on day 6 p.i., precisely towards the superior colliculus. The brain sections also showed that EBD was present in the thalamus and around the hypothalamic paraventricular nucleus. Moreover, western blot (WB) with Claudin-5, a tight junction protein, showed a significant decrease of its expression on day 6 p.i. (Fig. 7d, bottom). Altogether, this data indicates that rHSV-1 causes BBB disruption that can be rapidly evaluated by an intraperitoneal CTZ injection.

## Discussion

Our study describes the development of a neurovirulent rHSV-1 expressing fluorescent tdTomato protein and Gluc enzyme, which constitutes a promising tool to study HSE pathogenesis. Many research groups have edited the HSV-1 genome for decades by traditional viral genome editing strategies such as chemical mutagenesis, site-directed mutagenesis by HDR, overlapping cosmid clones and the BAC system. These approaches can generate infectious HSVs used for studying gene function and mutations^31–33^. To our knowledge, the generation and characterization of a neurovirulent rHSV-1 obtained by genetically modifying a clinical strain have not been described yet.

BAC technology provides a way to generate recombinant HSVs. However, this system remains challenging because of the requirement of cloning the large HSV-1 genome into a BAC vector. In many studies, newly-identified mutations in clinical HSV-1 isolates are inserted into the BAC-cloned genome of a laboratory strain, rather than cloning the whole genome of the new clinical strain with novel mutations into the BAC^34^. Moreover, the insertion of long vectors like BACs usually results in phenotypic changes, including altered viral growth and even a neurovirulence attenuation, which is problematic for HSE studies^35^. One proposed solution to avoid the loss of neurovirulence was to excise loxP sites flanking the BAC by Cre recombinase^36^. The targeted genomic region chosen to introduce exogenous fragments should be well characterized to limit HSV-1 neurovirulence attenuation. In our study, we decided to target the UL26-UL27 intergenic region of the clinical neurovirulent HSV-1 strain H25 to introduce two reporter genes. This location was already defined as a safe region to modify, with no effect on adjacent genes expression^13,37^. However, another recent study claimed that targeting the UL26-UL27 non-coding region for genome insertions could reduce viral replication^14^. The replicative capacity of our rHSV-1 suggested that the insertion of exogenous fragment into this location in HSV-1 strain H25 did not affect in vitro viral replication. Moreover, we observed significantly higher viral titers in the CNS of rHSV-1-infected BALB/c mice on day 6 p.i. Altogether, this data indicates that the impact of the insertion of exogenous DNA on neurovirulence depends on the strain that we modify and the exact location of the insert.

Conventional viral genome engineering methods are based on HDR in E. coli with HSV-1 genome cloned into a BAC or a virally-infected cell line. However, because of the low frequency of the HDR, the efficiency of modifications is reduced. Our study took advantage of the increased frequency of HDR triggered by gRNA-2/Cas9 induced DSB to insert the exogenous fragment encoding two reporter proteins. On the other hand, the off-target activity of gRNA/Cas9 resulting in unwanted random mutations is a potential limitation^38^. Based on that, we sequenced both rHSV-1 and clinical HSV-1 strain H25 (WT) to evaluate whether off-target, and other random mutations occurred during the production and the isolation of rHSV-1. Comparative genomic analysis of the two strains by next generation sequencing (NGS) revealed 3 non-silent mutations within three different genes. We suggest that these three non-silent mutations appeared randomly during rHSV-1 purification passages^39^. In addition, there were no phenotypic changes for rHSV-1, except an increase in neurovirulence, which could be related to Thr461Ile substitution in UL41 gene encoding vhs. We believe that such vhs mutation can alter its activity and may affect viral clearance by modulating the inflammatory response^40,41^.

By inserting two reporter genes (encoding for Gaussia Luciferase and tdTomato), we aimed to produce rHSV-1 generating bioluminescence and fluorescence that would be used for in vivo imaging of infected brains during HSE by BLI and bi-photonic fluorescent microscopy^42^. The tdTomato reporter was used for viral distribution studies on brain sections without immunostaining. We observed similar distribution patterns, elicited by rHSV-1 and WT HSV-1, in BALB/c mouse brains. Interestingly, the tdTomato signal was absent on some of the GFP^+^ immunostained rHSV-1-infected brain cells. We believe that this phenomenon may be explained by slight amounts of tdTomato, compared to a large quantity of HSV-1 proteins immunostained by polyclonal antibodies. The location of the insert within the UL26-UL27 intergenic region classified as a late-transcribed gene could also lead to a late production of this fluorescent protein. However, the activity of Gluc encoded by the same insert was detected as early as 12 h p.i., suggesting that even late gene (γ) transcription starts very early throughout infection as reported previously^43^.

Our new tool was designed to overcome many of the problems that researchers experience with HSE animal studies performed with conventional techniques. Traditional methods requiring animal sacrifices provide information for a specific time point. BLI of rHSV-1-infected mice allows studying the time course of the infection on the same animals, reducing the number of required animals and avoiding animal-to-animal variability. Before validating rHSV-1 kinetics in vivo, we characterized it on Vero cells. Secreted Gluc activity in infected cell culture supernatants highly correlated with viral titers, suggesting that rHSV-1 could be used as an efficient in vitro antiviral screening tool. Next, we analyzed Gluc activity in different biological liquids collected from mice, looking for a correlation with viral titers in the CNS. Similar to previous studies on brain tumors expressing Gluc, significant correlations were obtained, indicating that a simple luminescence analysis of plasma samples could be used to estimate viral titers in the brains^44,45^. As a proof-of-concept, the antiviral activity of VACV for HSE was evaluated in vivo by Gluc activity in plasma of rHSV-1-infected mice. Our in vivo antiviral study also exhibited a correlation between viral titers in brain and luminescence levels in plasma. Reduced viral titers on both days 4 and 6 p.i. in VACV-treated mice led to significantly increased survival rates.

The combination of IVIS images and immunofluorescence studies on brain sections helps identify potential dissemination routes of HSV-1 in HSE, as performed for other viruses^46,47^. According to our observations, following intranasal infection, rHSV-1 first infiltrates the brain via olfactory nerves, then uses the main olfactory route to infect the hypothalamus, including the suprachiasmatic nucleus (SCN). Thereafter, rHSV-1 can reach the optic nerve and induce conjunctivitis. The virus subsequently migrates to different parts of the diencephalon and mesencephalon. It has already been shown that bioluminescence intensity analysis could be used to estimate infectious virus titers^48^. We were also able to assess viral titers in the CNS of rHSV-1 infected mice using radiance values obtained from BLI following intravenous CTZ injection. Intraperitoneal CTZ injection revealed Gluc accumulation in different peripheral organs, suggesting that Gluc produced in the CNS can easily cross the BBB on day 6 p.i. Furthermore, the bioluminescent signal emitted from the CNS following intraperitoneal CTZ injection showed that CTZ can also cross the BBB. Based on these two observations, we assumed that the BBB was altered during the HSV-1 infection. Additionally, EBD assay on rHSV-1-infected mice revealed a significant increase of BBB permeability on day 6 p.i., compared to day 0 and day 4 p.i. We also confirmed BBB disruption by analyzing Claudin-5 expression in whole brain extracts on days 0, 4 and 6 p.i. There was a significant reduction in Claudin-5 levels on day 6 p.i., suggesting no selective exchange between the CNS and blood compartments.

An important limitation of BLI is a low spatial resolution that makes the identification of the exact location of infected areas more difficult, especially in the CNS, because of limited substrate bioavailability caused by the BBB and reduced amount of emitted light by skin and skull. However, rHSV-1 demonstrated significant advantages over conventional methods used to assess antiviral drugs in HSE by allowing to monitor CNS infection with less or non-invasive samples using bioluminescence. Our strategy highlights the feasibility of developing new recombinant HSV-1 with reporter proteins and without neurovirulence attenuation by using the CRISPR-Cas9 system on clinical strains. This new rHSV-1 reporter strain is a useful tool to improve our knowledge of HSE pathogenesis and to simplify the assessment of therapeutic modalities.

## Materials and methods

### Viruses and cells

The clinical neurovirulent HSV-1 strain H25 was used to generate rHSV-1. Vero cells (ATCC #CCL-81) were cultured in minimal essential medium (MEM) (Gibco/Invitrogen) supplemented with 10% fetal bovine serum (FBS) (Wisent) and 1% HEPES (4-(2-hydroxyethyl)-1-piperazineethanesulfonic acid), at 37 °C in 5% CO_2_. Viral titers were measured by plaque assays, as described elsewhere^49^.

### Mice

Six-week-old male BALB/c mice were obtained from Charles River. Mice were slightly anesthetized and infected with clinical HSV-1 strain H25 or rHSV-1 in 20 µL of MEM by intranasal inoculation. Animals were monitored three times daily for 14 days and sacrificed when body weight loss ≥ 20% or two other obvious sickness signs were observed. All animals were used in accordance with the Canadian Council on Animal Care guidelines, and the protocol was approved by the Animal Care Ethics Committee of Laval University (protocol no. 2017072). The study was carried out in compliance with the ARRIVE guidelines.

### Guide RNAs (gRNAs) design and plasmid construction

The UL26-UL27 intergenic region of HSV-1 strain H25 was chosen to insert the exogenous fragment. gRNAs were designed using the CHOPCHOP web tool (https://chopchop.cbu.uib.no) and using the HSV-1 strain 17 complete genome (Sequence ID: JN555585.1)^50^. Four gRNAs were cloned into the eSpCas9(1.1) PX330-like plasmid (Addgene). Three gRNAs target the UL26-UL27 intergenic region, and one did not target the selected region and was used as a negative control. The eSpCas9 plasmids were constructed as described elsewhere^51^.

The CRISPR-Cas9-induced HDR strategy was used to insert a 2918 bp exogenous fragment into the viral genome. The donor plasmid was constructed by cloning two gBlocks (produced by Integrated DNA Technologies) into the pEGFP-N1 vector used as a backbone plasmid. gBlocks-1 and 2 were digested with EcoRI/NotI and NotI/KpnI restriction enzymes (New England Biolabs (NEB)), respectively. Digested fragments were then ligated into the respective restriction sites of the pEGFP-N1 vector in two separate steps. Next, 128 pb left, and 150 pb right homology arms were amplified and digested respectively with AseI and NotI enzymes. Digested PCR products were ligated in the respective restriction sites of the donor plasmid in two separate steps.

### gRNA selection and indel mutation analysis

80–90% confluent Vero cells seeded in 6-well plates were transfected with 2 µg of gRNA/eSpCas9 plasmid. 12 h post-transfection, cells were infected with HSV-1 strain H25 at a MOI of 1. After 90 min of incubation, the medium was gently aspirated. Infected cells were washed with phosphate-buffered saline (PBS) and overlayed with MEM containing 2% FBS. To analyze the % of gRNA-mediated-NHEJ, supernatants containing a mixture of WT and mutant viruses were collected at 36 h p.i. Diluted supernatants were used to infect Vero cells in 24-well plates. Genomic DNA extractions were performed with QIAamp DNA Blood Mini Kit (Qiagen) on day 3 post-infection. The UL26-UL27 intergenic region of the virus mixture was amplified by Q5 Hot Start High-Fidelity DNA polymerase (NEB). PCR products were analyzed on a 0.8% agarose gel. The bands of interest around 1500 bp in size were gel-purified, and the first 260 bp of 5' end of amplicons were analyzed by Sanger sequencing. Multiple sequence alignments using ClustalW were performed to evaluate gRNAs by verifying deletions and/or insertions of nucleotides into the UL26-UL27 intergenic region^52^. The ‘Inference of CRISPR Edits’ (ICE) analysis was performed to assess gRNA editing efficiency (https://www.synthego.com/products/bioinformatics/crispr-analysis).

### Generation and isolation strategy for rHSV-1

The transfection/infection method was used to generate the recombinant virus by introducing reporter genes under the control of the CMV MIE promoter into the intergenic region of UL26-UL27 via HDR, as described previously^13^. Cells were transfected with 2 µg of a mixture of plasmids (gRNA and donor plasmid/1:1 ratio) and Lipofectamine 2000 (Life Technologies) (3 μL/μg DNA) in 1 mL of OptiMEM (Gibco, Thermo Fisher Scientific) for 12 h and then infected with WT HSV-1 strain H25. For the isolation of the recombinant virus, tdTomato^+^ cells were visualized by fluorescence microscopy (Nikon—Eclipse TE300 Inverted Microscope). Picked cells were resuspended in a cell culture medium and used to infect new Vero cells. Five rounds of plaque purification were performed to purify the recombinant virus before analysis.

### Multiplex PCR targeted amplicon (MTA-Seq) and whole genome sequencing

Before the characterization of rHSV-1, we confirmed by Illumina deep sequencing that viral isolation and production steps were successfully performed without any remaining WT HSV-1 contaminant. A PCR assay followed by deep sequencing of its products was developed to identify heterogeneous viral populations, as described elsewhere^53,54^. We designed a multiplex PCR that combines a common forward primer for both and two specific reverse primers targeting WT or recombinant HSV-1. Viral DNA was isolated with the Blood-Mini-Kit (Qiagen). Q5 Hot Start High-Fidelity DNA polymerase (NEB) was used to amplify 584 bp and 543 bp fragments for WT and recombinant virus, respectively. The bands of interest (between 500 and 650 bp) were gel-purified and then quantified by Qubit 3.0 (Thermo Fisher Scientific) to adjust the concentration before Illumina deep sequencing.

In addition to PCR products, we also sequenced the whole genomes of WT and recombinant viruses. DNA extracts were first quantified using Qubit 3.0. Then, 1 ng of each sample was used to prepare DNA libraries using the Nextera XT sample preparation kit (Illumina), according to the manufacturer's instructions. A purification step with AMPure XP beads (Beckman Coulter) to remove very short library fragments was performed prior to library normalization. The libraries were then multiplexed, clustered, and finally sequenced using the Nextera XT kit (Illumina) as described by the manufacturer, except that 10% phiX Control (Illumina) was added to the library pool. The paired-end sequencing (2 × 250 nucleotides [nt]) was performed on a MiSeq system (Illumina).

Reads were first demultiplexed using bcl2fastq v2.20. WT HSV-1 and rHSV-1 reads were independently aligned with HSV-1 strain 17 genome (LT576870.1) using Snippy v4.3.6 (https://github.com/tseemann/snippy). Using Tablet v1.19.09.03, coding regions with low to no coverage were manually identified, and PCR primers were designed to amplify and sequence these regions using Sanger sequencing^55^. The resulting Sanger sequences and Illumina reads were used to assemble the genomes using Spades genome assembler v3.13.0^56^. After assembly, reads from rHSV-1 were aligned with WT HSV-1 contigs in order to find possible mutations using Snippy.

Reads corresponding to the multiplexed PCR of the insertion junction were aligned to the contigs from rHSV-1 containing the expected insertion and to the complete genome of HSV-1 strain 17. Coverage at each position was retrieved from the bam files using Python and PySam v0.16.0.1 (https://github.com/pysam-developers/pysam).

### Off-target analysis for gRNAs

The initial off-target analysis was performed on CHOPCHOP while designing gRNAs. Before that, UL26-UL27 intergenic region of HSV-1 strain H25 was amplified, then Sanger sequenced. A similar HSV-1 genome (JN555585.1), also available in the reference genomes database of CHOPCHOP, was identified by the UL26-27 sequence of HSV-1 strain H25. Our initial in silico analysis of UL26-UL27 fragment permitted to choose three gRNAs with no potential off-targets on the reference genome (JN555585.1). Another reference genome (LT576870.1), 99.2% identical to JN555585.1, was used for WGS analysis of HSV-1 H25. As these three HSV-1 genomes were highly similar (> 99%), we concluded that there were no potential off-target sites for our gRNAs on the genome of HSV-1 H25 strain. In addition, gRNA sequences were used to find sequences from the WT reads containing possible target for CRISPR-Cas9, by using BLAST (blastn-short). All 96 matching reads were aligned against the reference sequence (LT576870.1). No possible off-targets were detected using this analysis. Considering the three analyses we performed; namely CHOPCHOP on JN555585.1, gRNA blast on LT576870.1 and gRNA homology search on HSV1-WT, we concluded that no off-targets were possible for the selected gRNA.

### Characterization of tdTomato expression

We first observed the expression of tdTomato in vitro while isolating rHSV-1. tdTomato expression was confirmed on infectious spots of rHSV-1 with immunofluorescence assays (IF). Additionally, Vero cells on cover slips were infected with isolated rHSV-1, then also immunolabeled to visualize tdTomato expression in a single cell level. The following approach was performed for both immunofluorescence assay. Infected Vero cells were fixed 72 h.p.i. by adding 500 µL of 4% paraformaldehyde (PFA) (30 min at room temperature (RT)). After 3 washes with 1X PBS, cells were first permeabilized by adding 500 µL of 0.1% Triton X-100 in 1X PBS (15 min at RT), then blocked with 500 µL of 2% bovine serum albumin in PBS (60 min at RT). Cells were incubated with HSV-1/2 polyclonal primary antibody (Bio-Rad Laboratories) for 2 h, then with secondary Alexa Fluor 488-labeled goat IgG (Invitrogen/Thermo Fisher Scientific) antibody for 45 min at RT protected from light. After three washes with 1X PBS, a fluorescent microscopy image (Fig. 2b) was captured using a Nikon fluorescence microscope (Eclipse TE300 Inverted Microscope). Image acquisition of rHSV-1-infected Vero cells (Fig. 2c) was performed (Z-stack of 11 images at 0.2-µM intervals) with an Olympus IX80 equipped with a WaveFX-Boreal-SC Yokagawa spinning disk confocal (Quorum Technologies) and an Orca Flash4.0 camera (Hamamatsu).

Harvested brains were prepared for the immunofluorescence analysis as described elsewhere^57^. Brain sections fixed with PFA 4% were incubated with the same antibodies as mentioned above. Finally, nuclear staining was performed using DAPI. Confocal fluorescence microscopy images were captured using an Axio Imager M2 epifluorescence microscope equipped with an AxioCamMRm (Carl Zeiss Microscopy GmbH).

### In vitro analysis of viral replicative capacities and Gluc activity

Replication kinetics of rHSV-1 were analyzed by two in vitro approaches: (1) RTCA system (xCELLigence; ACEA Biosciences, Inc.) and (2) plaque assay*.* The RTCA system is a real-time monitoring technology measuring the electronic impedance (referred to as the cell index (CI)), using gold microelectrode sensor arrays integrated in a special cell culture plate. The quality of cell–cell and cell-microelectrode interactions, the number of cells in the well and the overall cell morphology are factors affecting the CI values measured to determine viral replicative capacities. Confluent Vero cells seeded in E-Plates 96 (6-wells per time point) were inoculated with WT or rHSV-1 at a MOI of 0.01 and incubated for 90 min. The viral suspension was removed and replaced by fresh culture medium (MEM plus 2% FBS). Normalized CI (cell index) values were then recorded every 30 min for 72 h, as described elsewhere^58^.

To confirm RTCA results, we compared WT and rHSV-1 replicative capacities by plaque assay. As mentioned above, confluent Vero cells seeded in 24-well plates were infected with WT or rHSV-1 at a MOI of 0.01. Briefly, cell culture supernatants from triplicate wells were sampled every 8 or 12 h for 54 h. 100 μL of collected supernatants were used to measure viral titers. Simultaneously, 30 μL of the supernatant were used to determine the Gluc activity by the Gluc reporter-based assay as described elsewhere^59^.

### In vivo analysis of neurovirulence, viral replicative capacity and Gluc activity

BALB/c mice were infected with 1500 PFUs of clinical HSV-1 strain H25 or rHSV-1, and survival rates were compared. Luciferase activity in brain homogenates, whole blood, plasma and urine collected from rHSV-1-infected mice on days 0, 4 and 6 p.i. was measured. Brain homogenates were centrifuged at 10,000×*g* for 10 min at 4 °C. Blood samples (~ 150 μL) were withdrawn from the facial vein of mice and collected in EDTA-coated tubes (Thermo Fisher Scientific) to prevent coagulation. For plasma isolation, half of the blood samples was centrifuged for 10 min at 1 500×*g* at 4 °C. Urine samples were collected by abdominal palpation. Then, blood, plasma or urine samples were used to carry out luciferase assay^45^. Except for urine samples, 30 μL of undiluted samples were transferred to flat solid bottom and opaque-walled white 96-well laminator Costar plates (Fisher Scientific Inc). For urine sample analysis, 15 μL of urine samples were mixed with 15 μL of PBS 1X.

### In vivo imaging and monitoring of HSE

rHSV-1-infected mice were anesthetized with 2.5% vaporized isoflurane inhalation. Animals were monitored by BLI resulting from Gluc activity. To validate our HSE model, 50 μL of Inject-A-Lume coelenterazine substrate diluted in Fuel-Inject (Nanolight Technology, Prolume Ltd.) was injected intravenously (i.v.) through the tail vein. Two min after CTZ administration, mice were imaged (60 s exposure time) with an IVIS Spectrum in vivo imaging system (Perkin Elmer). For BBB permeability analysis, the same amount of CTZ was injected intraperitoneally (i.p.), and 6 images were acquired at 3 min-intervals for 15 min to monitor bioluminescent signal. Data [in radiance (photons per second per square centimeter per steradian (p/s/cm2/sr)] were analyzed using Living Image 3.0 software (Perkin Elmer/Caliper Life Sciences).

### Evaluation of valacyclovir treatment by bioluminescence

Susceptibility of WT and rHSV-1 to acyclovir (Sigma-Aldrich) was first tested by PRA^58^. The effect of valacyclovir (Sigma-Aldrich) treatment on HSE was evaluated by measuring bioluminescence signal resulting from Gluc activity in plasma samples. Mice were infected intranasally with 2.5 × 10^3^ PFU of HSV-1 strain H25. VACV (1 mg/mL in drinking water ad libitum from day 3 and on) was administered to infected mice, as described elsewhere^26^. For survival rate experiments, mice were monitored for 14 days for the appearance of HSE-related signs or death as described above. Subsets of mice were sacrificed before the infection and on days 4 and 6 p.i. Brain homogenates were prepared to quantify viral titers, as reported elsewhere^57^.

### Evaluation of BBB disruption

The Evans blue dye (EBD) test was used to assess the blood–brain barrier (BBB) integrity. Non-infected control and mice infected intranasally with 1500 PFU were given 50 μL of EBD i.v. [1% (w/v) in PBS] (Sigma) on days 0, 4 and 6 p.i. Three hours later, mice were perfused with 20 mL PBS 1X and brains were harvested and photographed to analyze EBD-stained areas visually. Western blotting was conducted by using total protein extract of the brain to detect Claudin-5. Total cellular protein was extracted by sonicating the brain in chilled Cell Lysis buffer (Sigma) followed by centrifugation at 11, 000×*g* for 20 min, and then the soluble supernatant fraction was collected. An equal amount of total protein (50 µg) was separated by SDS-PAGE, transferred onto nitrocellulose membrane and incubated overnight with polyclonal antibodies against claudin-5 (Santa-Cruz Biotechnologies Inc.). Simultaneously, another membrane was prepared and incubated with polyclonal antibodies against β-catenin (Abcam) selected as a housekeeping protein. Following incubation with secondary antibodies conjugated with horseradish peroxidase (HRP), chemiluminescence assay was performed with Supersignal West Pico chemiluminescent substrate (Thermo Fisher Scientific). Uncropped images of scanned western blots shown in (d) are provided (Supplementary Fig. 3).

### Statistical analyses

All statistical analyses, regression and correlation studies were performed using GraphPad Prism version 8.4.2, GraphPad Software, San Diego, California USA (www.graphpad.com), after excluding outliers by Grubb's test. A P value of < 0.05 was considered statistically significant. All data are presented as means ± standard errors of the mean (SEM). EC50s of acyclovir against two viruses were compared by the Mann–Whitney test. Survival rates were analyzed by log-rank Mantel-Cox tests. All other data were analyzed by two-way ANOVA with Sidak's multiple comparisons test.

## Supplementary Information

Supplementary Figure 1.

Supplementary Figure 2.

Supplementary Figure 3.

## Data Availability

The data and primer sequences that support the findings of this study are available upon request from the corresponding author. The genomic data is available through Biosample database at NCBI (accession numbers: SAMN18751846 (HSV-1 strain H25) and SAMN18751847 (rHSV-1)).
