# Supplementary material for: A novel bioluminescent herpes simplex virus 1 for in vivo monitoring of herpes simplex encephalitis
Source: Sci Rep. 2021 Sep 21;11:18688. doi: 10.1038/s41598-021-98047-z (PMC8455621; doi:10.1038/s41598-021-98047-z)
Supplement: Supplementary file 1 — Supplementary Figure 1. [file 41598_2021_98047_MOESM1_ESM.pdf]

Title Page

A novel bioluminescent herpes simplex virus 1 for *in vivo* monitoring of herpes simplex encephalitis

Olus Uyar[1], Pier-Luc Plante[2], Jocelyne Piret[1], Marie-Christine Venable[1], Julie Carbonneau[1], Jacques Corbeil[2], and Guy Boivin[1*]

[1]Research Center in Infectious Diseases, CHU de Québec- Laval University Research Center and Department of Pediatrics and Microbiology, Faculty of Medicine, Laval University, Quebec City, QC, Canada

[2]Research Center in Infectious Diseases, CHU de Québec- Laval University Research Center and Department of Molecular Medicine and Big Data Research Centre, Faculty of Medicine, Laval University, Quebec City, QC, Canada

a

Name - gBlock EcoRI gBlocks® Gene Fragments 1369 base pairs

```
5'- CCT GCT GAA TTC TAA TGG GAG TCA AAG TTC TGT TTG CCC TGA TCT GCA TCG CTG TGG CCG AGG CCA AGC
CCA CCG AGA ACA ACG AAG ACT TCA ACA TCG TGG CCG TGG CCA GCA ACT TCG CGA CCA CGG ATC TCG ATG CTG
ACC GCG GGA AGT TGC CCG GCA AGA AGC TGC CGC TGG AGG TGC TCA AAG AGA TGG AAG CCA ATG CCC GGA AAG
CTG GCT GCA CCA GGG GCT GTC TGA TCT GCC TGT CCC ACA TCA AGT GCA CGC CCA AGA TGA AGA AGT TCA TCC
CAG GAC GCT GCC ACA CCT ACG AAG GCG ACA AAG AGT CCG CAC AGG GCG GCA TAG GCG AGG CGA TCG TCG ACA
TTC CTG AGA TTC CTG GGT TCA AGG ACT TGG AGC CCA TGG AGC AGT TCA TCG CAC AGG TCG ATC TGT GTG TGG
ACT GCA CAA CTG GCT GCC TCA AAG GGC TTG CCA ACG TGC AGT GTT CTG ACC TGC TCA AGA AGT GGC TGC CGC
AAC GCT GTG CGA CCT TTG CCA GCA AGA TCC AGG GCC AGG TGG ACA AGA TCA AGG GGG CCG GTG GTG ACG GAA
GCG GAG CTA CTA ACT TCA GCC TGC TGA AGC AGG CTG GAG ACG TGG AGG AGA ACC CTG GAC CTA TGG TGA GCA
AGG GCG AGG AGG TCA TCA AAG AGT TCA TGC GCT TCA AGG TGC GCA TGG AGG GCT CCA TGA ACG GCC ACG AGT
TCG AGA TCG AGG GCG AGG GCG AGG GCC GCC CCT ACG AGG GCA CCC AGA CCG CCA AGC TGA AGG TGA CCA AGG
GCG GCC CCC TGC CCT TCG CCT GGG ACA TCC TGT CCC CCC AGT TCA TGT ACG GCT CCA AGG CGT ACG TGA AGC
ACC CCG CCG ACA TCC CCG ATT ACA AGA AGC TGT CCT TCC CCG AGG GCT TCA AGT GGG AGC GCG TGA TGA ACT
TCG AGG ACG GCG GTC TGG TGA CCG TGA CCC AGG ACT CCT CCC TGC AGG ACG GCA CGC TGA TCT ACA AGG TGA
AGA TGC GCG GCA CCA ACT TCC CCC CCG ACG GCC CCG TAA TGC AGA AGA AGA CCA TGG GCT GGG AGG CCT CCA
CCG AGC GCC TGT ACC CCC GCG ACG GCG TGC TGA AGG GCG AGA TCC ACC AGG CCC TGA AGC TGA AGG ACG GCG
GCC ACT ACC TGG TGG AGT TCA AGA CCA TCT ACA TGG CCA AGA AGC CCG TGC . . . -3'
```

Name - gBlock NotI gBlocks® Gene Fragments 1016 base pairs

```
5'- CCT GCT GGT ACC GGC AGC ACC GGC AGC GGC AGC TCC GGC ACC GCC TCC TCC GAG GAC AAC AAC ATG GCC
GTC ATC AAA GAG TTC ATG CGC TTC AAG GTG CGC ATG GAG GGC TCC ATG AAC GGC CAC GAG TTC GAG ATC GAG
GGC GAG GGC GAG GGC CGC CCC TAC GAG GGC ACC CAG ACC GCC AAG CTG AAG GTG ACC AAG GGC GGC CCC CTG
CCC TTC GCC TGG GAC ATC CTG TCC CCC CAG TTC ATG TAC GGC TCC AAG GCG TAC GTG AAG CAC CCC GCC GAC
ATC CCC GAT TAC AAG AAG CTG TCC TTC CCC GAG GGC TTC AAG TGG GAG CGC GTG ATG AAC TTC GAG GAC GGC
GGT CTG GTG ACC GTG ACC CAG GAC TCC TCC CTG CAG GAC GGC ACG CTG ATC TAC AAG GTG AAG ATG CGC GGC
ACC AAC TTC CCC CCC GAC GGC CCC GTA ATG CAG AAG AAG ACC ATG GGC TGG GAG GCC TCC ACC GAG CGC CTG
TAC CCC CGC GAC GGC GTG CTG AAG GGC GAG ATC CAC CAG GCC CTG AAG CTG AAG GAC GGC GGC CAC TAC CTG
GTG GAG TTC AAG ACC ATC TAC ATG GCC AAG AAG CCC GTG CAA CTG CCC GGC TAC TAC TAC GTG GAC ACC AAG
CTG GAC ATC ACC TCC CAC AAC GAG GAC TAC ACC ATC GTG GAA CAG TAC GAG CGC TCC GAG GGC CGC CAC CAC
CTG TTC CTG TAC GGC ATG GAC GAG CTG TAC AAG TGA CTG TGC CTT CTA GTT GCC AGC CAT CTG TTG TTT GCC
CCT CCC CCG TGC CTT CCT TGA CCC TGG AAG GTG CCA CTC CCA CTG TCC TTT CCT AAT AAA ATG AGG AAA TTG
CAT CGC ATT GTC TGA GTA GGT GTC ATT CTA TTC TGG GGG GTG GGG TGG GGC AGG ACA GCA AGG GGG AGG ATT
GGG AAG ACA ATA GCA GGC ATG CTG GGG ATG CGG TGG GCT CTA TGG GCG GCC GCT GCA TGA TCT ACG TGC GTC
ACA TGC AGT AC -3'
```

b

gBlocks Sequence Map	
gBlock-1 (EcoRI) - position	**IDENTITY**
1--13	portion with EcoRI site
14--15	Extra nucleotides for ORF correction
16--569	Gaussia Luciferase
570--635	2A Self-cleaving peptide coding sequence
636--1321	tdTomato 1
1322-1369	portion with NotI site
gBlock-2 (NotI) - position	**IDENTITY**
1--9	portion with NotI site
10--753	tdTomato 2
754--979	Bovine Growth Hormon terminator signal + PolyA sequence
987--989	Modified protospacer adjacent motif
990--1016	portion with KpnI site

c

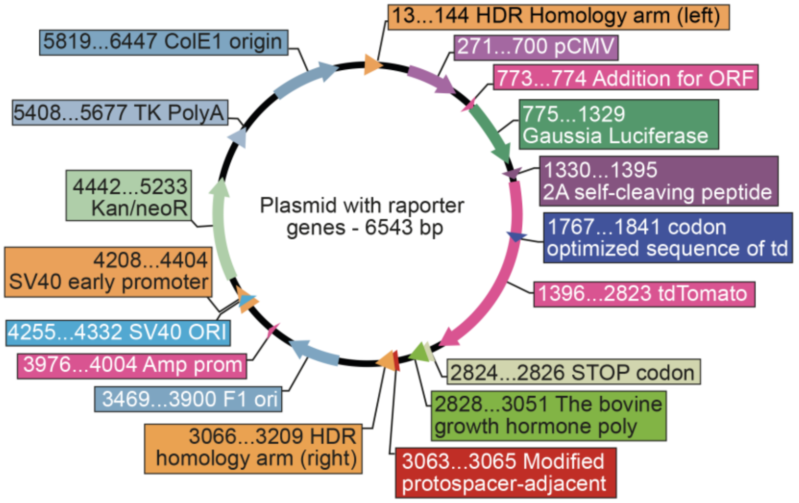

Supplementary Figure 1: (a) gBlock gene fragments, (b) maps and (c) the donor plasmid map. Serial Cloner 2.6 (http://serialbasics.free.fr/Serial_Cloner.html) was used to analyze sequence data, and design the donor plasmid map (c).
