# Supplementary material for: A novel bioluminescent herpes simplex virus 1 for in vivo monitoring of herpes simplex encephalitis
Source: Sci Rep. 2021 Sep 21;11:18688. doi: 10.1038/s41598-021-98047-z (PMC8455621; doi:10.1038/s41598-021-98047-z)
Supplement: Supplementary file 2 — Supplementary Figure 2. [file 41598_2021_98047_MOESM2_ESM.pdf]

Title Page

A novel bioluminescent herpes simplex virus 1 for *in vivo* monitoring of herpes simplex encephalitis

Olus Uyar[1], Pier-Luc Plante[2], Jocelyne Piret[1], Marie-Christine Venable[1], Julie Carbonneau[1], Jacques Corbeil[2], and Guy Boivin[1*]

[1]Research Center in Infectious Diseases, CHU de Québec- Laval University Research Center and Department of Pediatrics and Microbiology, Faculty of Medicine, Laval University, Quebec City, QC, Canada

[2]Research Center in Infectious Diseases, CHU de Québec- Laval University Research Center and Department of Molecular Medicine and Big Data Research Centre, Faculty of Medicine, Laval University, Quebec City, QC, Canada

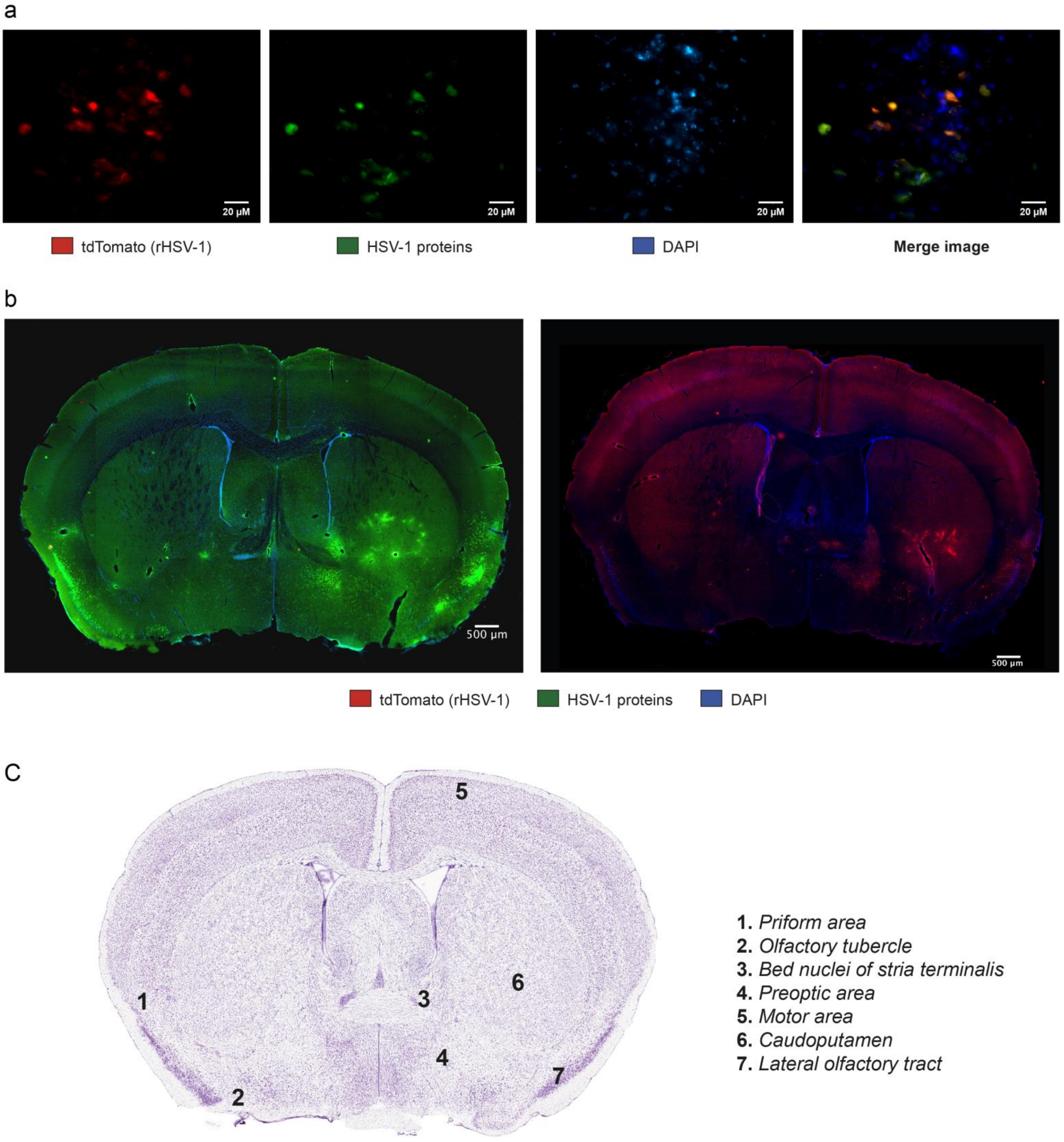

Supplementary Figure 2: Distribution patterns of WT HSV-1 and rHSV-1 infectious spots in the CNS are similar. (a) Colocalization of tdTomato signal (red) with HSV-1 staining (green) in rHSV-infected CNS cells (Scale bar = 20 μm) (b) Representative brain sections illustrating the localization of HSV-1 proteins (upper left, green) and tdTomato expressed by rHSV-1 (upper right, red) in different regions of the brain on day 6 post-infection. Brain sections from WT HSV-1-infected mouse were immunostained with a primary polyclonal rabbit anti-HSV-1/2 antibody and a secondary Alexa 488-conjugated goat anti-rabbit antibody (green), followed by staining with DAPI (blue). rHSV-1-infected brain sections were only stained with DAPI (blue). (c) Representative coronal section (P56) of the mouse brain (lower left) showing infected regions on day 6 p.i. (Image credit: Allen Institute). HSV-1+ brain areas are indicated with numbers 1 to 7 (lower right)
