# Supplementary material for: A novel bioluminescent herpes simplex virus 1 for in vivo monitoring of herpes simplex encephalitis
Source: Sci Rep. 2021 Sep 21;11:18688. doi: 10.1038/s41598-021-98047-z (PMC8455621; doi:10.1038/s41598-021-98047-z)
Supplement: Supplementary file 3 — Supplementary Figure 3. [file 41598_2021_98047_MOESM3_ESM.pdf]

Title Page

A novel bioluminescent herpes simplex virus 1 for *in vivo* monitoring of herpes simplex encephalitis

Olus Uyar[1], Pier-Luc Plante[2], Jocelyne Piret[1], Marie-Christine Venable[1], Julie Carbonneau[1], Jacques Corbeil[2], and Guy Boivin[1*]

[1]Research Center in Infectious Diseases, CHU de Québec- Laval University Research Center and Department of Pediatrics and Microbiology, Faculty of Medicine, Laval University, Quebec City, QC, Canada

[2]Research Center in Infectious Diseases, CHU de Québec- Laval University Research Center and Department of Molecular Medicine and Big Data Research Centre, Faculty of Medicine, Laval University, Quebec City, QC, Canada

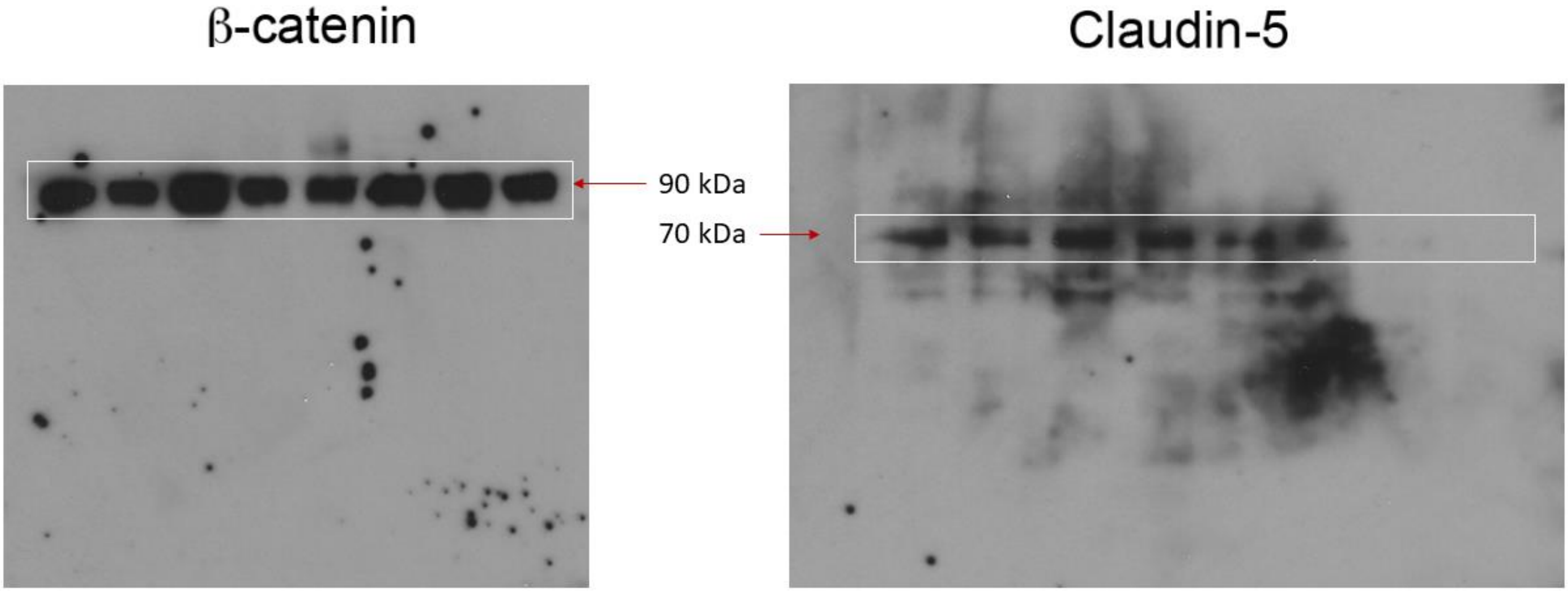

Uncropped images of scanned western blots shown in Fig.7(d) are provided.
